# Passive immunization with equine RBD-specific Fab protects K18-hACE2-mice against Alpha or Beta variants of SARS-CoV-2

**DOI:** 10.3389/fimmu.2022.948431

**Published:** 2022-08-15

**Authors:** Mariette Barbier, Katherine S. Lee, Mayur S. Vikharankar, Shriram N. Rajpathak, Nandkumar Kadam, Ting Y. Wong, Brynnan P. Russ, Holly A. Cyphert, Olivia A. Miller, Nathaniel A. Rader, Melissa Cooper, Jason Kang, Emel Sen-Kilic, Zeriel Y. Wong, Michael T. Winters, Justin R. Bevere, Ivan Martinez, Rachayya Devarumath, Umesh S. Shaligram, F. Heath Damron

**Affiliations:** ^1^ Department of Microbiology, Immunology, and Cell Biology, West Virginia University, Morgantown, WV, United States; ^2^ Vaccine Development Center at West Virginia University Health Sciences Center, Morgantown, WV, United States; ^3^ Research and Development Department, Serum Institute of India Pvt. Ltd., Pune, India; ^4^ Savitribai Phule Pune University, Pune, India; ^5^ Research and Development Department, Isera Biological Pvt. Ltd., Pune, India; ^6^ Department of Biological Sciences, Marshall University, Huntington, WV, United States; ^7^ School of Medicine, West Virginia University Cancer Institute, Morgantown, WV, United States; ^8^ Department of Molecular Biology and Genetic Engineering, Vasantdada Sugar Institute, Pune, India

**Keywords:** SARS-CoV-2, COVID-19, Equine F(ab’)_2_, EpF(ab’)_2_, passive immunization, polyclonal antibodies, K18-hACE2 transgenic mice, variant of concern (VOC)

## Abstract

Emergence of variants of concern (VOC) during the COVID-19 pandemic has contributed to the decreased efficacy of therapeutic monoclonal antibody treatments for severe cases of SARS-CoV-2 infection. In addition, the cost of creating these therapeutic treatments is high, making their implementation in low- to middle-income countries devastated by the pandemic very difficult. Here, we explored the use of polyclonal EpF(ab’)_2_ antibodies generated through the immunization of horses with SARS-CoV-2 WA-1 RBD conjugated to HBsAg nanoparticles as a low-cost therapeutic treatment for severe cases of disease. We determined that the equine EpF(ab’)_2_ bind RBD and neutralize ACE2 receptor binding by virus for all VOC strains tested except Omicron. Despite its relatively quick clearance from peripheral circulation, a 100μg dose of EpF(ab’)_2_ was able to fully protect mice against severe disease phenotypes following intranasal SARS-CoV-2 challenge with Alpha and Beta variants. EpF(ab’)_2_ administration increased survival while subsequently lowering disease scores and viral RNA burden in disease-relevant tissues. No significant improvement in survival outcomes or disease scores was observed in EpF(ab’)_2_-treated mice challenged using the Delta variant at 10μg or 100µg doses. Overall, the data presented here provide a proof of concept for the use of EpF(ab’)_2_ in the prevention of severe SARS-CoV-2 infections and underscore the need for either variant-specific treatments or variant-independent therapeutics for COVID-19.

## Introduction

In the fight against COVID-19, vaccination currently stands as the first line of defense against severe infections and deaths caused by the SARS-CoV-2 virus. In addition to vaccines, monoclonal antibody therapeutics have been used to treat cases of infection that are at risk of progressing to severe disease that may require hospitalization, and as prophylactic treatments for high-risk individuals post-exposure. Most of the vaccines and antibody treatments that are currently FDA-approved are based on the genome sequence of the original SARS-CoV-2 viral strain (Wuhan-1; ancestral) discovered in Wuhan, China in 2019. These immunotherapies target the receptor binding domain (RBD) of the viral spike protein which mediates host cell entry *via* the ACE2 receptor. RBD is one of two antigenic “supersites” (1) found on the spike protein and serves as a key target of neutralizing antibodies that develop in convalescent patients (2, 3). Since the initial discovery of SARS-Cov-2, numerous variants of concern (VOC) have appeared and spread throughout the world: B.1.1.7 or Alpha (isolated in the UK), B.1.351 or Beta (isolated in South Africa), B.1.1.248 or Gamma (isolated in Brazil), B.1.167.2 or Delta (isolated in India), and more recently B.1.1.529 or Omicron (isolated in South Africa). These strains are each characterized by an accumulation of both variant-specific and conserved mutations in RBD that mediate their resistance to binding and neutralization by host antibodies (4–7). Unfortunately, these mutations result in the decrease of approved therapeutic monoclonal antibody efficacy (4, 5, 7).

In the race to protect the global population against the rise of new VOCs, it is essential to design novel preventatives and therapeutics. In particular, neutralizing antibodies remain an attractive therapeutic option as they can interfere with viral replication by blocking the binding between RBD and the ACE2 receptor thus preventing entry into the cell. Novel approaches to designing SARS-CoV-2 therapeutic antibodies include the generation of antigen-binding fragments (F(ab’)_2_) rather than whole antibody. F(ab’)_2_ are produced through the enzymatic cleavage of immunoglobulins and conserve both the antigen-binding region and part of the hinge region. The use of monoclonal F(ab’)_2_ for the treatment of SARS-CoV-2 has already shown efficacious potential in various studies utilizing different methods to raise the particle (8–10). However, one important caveat of monoclonal antibodies that extends to most current F(ab’)_2_ is that they are designed to target one specific epitope, rendering treatment inefficacious in the event of mutation. To address this problem, F(ab’)_2_ can be prepared using a polyclonal hyperimmune equine serum. Equine polyclonal F(ab’)_2_ (EpF(ab’)_2_) have the advantage of reacting to multiple RBD epitopes, decreasing the potential for mutation-driven resistance to the therapy. For example, EpF(ab’)_2_ generated from equine antibodies against SARS-CoV-2 have already shown to possess ability to bind and neutralize the SARS-CoV-2 Gamma variant *in vitro* (11). In addition, EpF(ab’)_2_ can be produced at relatively low cost and production can easily be scaled up depending on animal availability, decreasing economic barriers and thus increasing the potential for utilization of this treatment in developing countries. Altogether, EpF(ab’)_2_ represent a promising approach for the treatment of SARS-CoV-2, however, additional studies are still needed to determine their therapeutic potential during infection, specifically against emerging VOC.

In this study, EpF(ab’)_2_ were produced by immunizing horses with a yeast-purified RBD HBsAg conjugate and tested in an *in vivo* preclinical model of SARS-CoV-2 challenge with K18-hACE2-transgenic mice (12–17). The EpF(ab’)_2_ utilized in this study were capable of binding and neutralizing SARS-CoV-2 *in vitro*. Mice were passively immunized using one of two different doses and challenged in studies utilizing a lethal challenge dose of Alpha, Beta, or Delta SARS-COV-2 variants. Despite their short half-life in serum as determined by serological kinetics studies, EpF(ab’)_2_ were able to protect K18-hACE2 mice against severe disease from Alpha and Beta, but not Delta VOC strains. Our work highlights the therapeutic potential of this technology and suggests improvements that can be made for broad application of the treatment against COVID-19.

## Materials and methods

### EpF(ab’)_2_ generation

The entire process of EpF(ab’)_2_ generation was carried out at Serum Institute of India Pvt.Ltd. SIIPL has pioneered an innovative VLP based recombinant vaccine that can stimulate the immune system to produce anti-RBD antibodies and thus protect against SARS-CoV-2 infection and COVID-19 disease. In a similar vein, SIIPL also developed RBD conjugate antigen for equine immunization. Indian horses (n=20) were immunized seven times (week 0, 2, 3, 4, 5, 6, and 7), with RBD-HBsAg VLP conjugate antigen (1mg/mL) *via* the subcutaneous route. Antigen quantity was increased over time in each subsequent vaccination: 200µg, 400µg, 800µg, 1200µg, 1600µg, 2000µg, except for the final vaccination at 1200µg. Freund’s adjuvant was used for the first four vaccinations at the following respective volumes: 0.250mL, 0.5mL, 1.0mL, 1.5mL. During the immunization period, plasma samples were taken (Isera Biological Pvt. Ltd) and analysed *via* ELISA for neutralizing antibody titres. At the end of the vaccination period, blood was collected for plasma purification as per regulatory guidelines (data not shown). Plasma (2-3L per animal) was purified *via* a fractionation process consisting of plasma dilution followed by pH adjustment with 5N HCL. Fractionation was carried out using pepsin for 1 hour followed by thermocoagulation processing. Caprylic acid was then slowly added to the digested plasma while stirring to precipitate any non-IgG proteins and centrifuged to separate the phases. SIIPL has developed an in-house chromatography method for further purification wherein the sample containing F(ab)2 antibodies and impurities (e.g. small peptides, high molecular weight aggregates, pepsin residue, caprylic acid and traces of albumin) are passed through a chromatography column containing selective resin. The purified sample is then subjected to ultrafiltration and diafiltration (TFF) steps to produce the usable Drug Substance (DS). The DS is stored between 2-8°C until use. The DS then underwent various analytical and stringent quality testing (data not shown).

### Acquisition of human convalescent sera

Serum was collected previously from a single patient at West Virginia University Hospital in early 2020 who was PCR positive for COVID-19 (IRB no. 2004976401) and used in previous studies by our lab (18). Serological analysis of the serum was performed in the WVUH clinical laboratory before transfer to the Vaccine Development Center. Serum was aliquoted into 1-ml cryovials and frozen at -80°Cto avoid freeze-thawing when used.

### SARS-CoV-2 peptide binding assay

Antibody binding of EpF(ab’)_2_, HCP, and sera pooled from Alpha SARS-CoV-2-challenged mRNA (BioNTech)-vaccinated mice to SARS-CoV-2 RBD regions was assessed using ELISA. Biotinylated RBD peptides spanning the neutralizing epitopes of the SARS-CoV-2 spike protein were prepared by ThermoFisher Scientific based on amino acid sequences from the RBD antigen. The RBD polypeptide sequence was analyzed by BepiPred 2.0 (19) and candidate epitopes were selected. The peptides were also compared to those used by Zhang, et al. (20) and 7 peptides were selected for the analysis. The sequences of the peptides are listed in **Table S1**. RBD peptides were diluted 4μg/100μL in 0.1% BSA/PBS (Bovine Serum Albumin: Research products international, A30075-250.0) in high-binding 96 well plate (Pierce 155500) and incubated shaking at room temperature for 1hr to coat. Plates were then washed 4 times with PBS-0.1%Tween20. Sample were then serially diluted 1:2 in 2% BSA/PBS starting at a concentration of 0.128mg/mL for (EpF(ab’)_2_. mRNA and HCP sera were diluted 1:20 in 2% BSA/PBS. After 1hr incubation shaking at room temperature, plates were washed 4 times with PBS-0.1%Tween20. Secondary antibody (Goat anti-mouse IgG secondary (Novus biological, NBP1-75130); Goat anti-equine IgG secondary (Novus biological, NB7421)) was added to all wells at a 1:2,000 dilution in 2% BSA/PBS, incubated shaking at room temperature for 1hr, then washed another 4 times with PBS-0.1%Tween20. 3,3’,5,5’-Tetramethylbenzidine (TMB) reagent (Biolegend, 421101) was added following the manufacturer’s instructions and incubated for 15min before the reaction was stopped with 50μL of 2N sulfuric acid. The assays were analyzed using the Synergy H1 plate reader at 450nm. Comparison of binding was measured using area under the curve analysis in GraphPad Prism v.9.0.0. Regions with highest binding to RBD peptides were represented on the 3D structure of RBD (Crystal structure Uniprot reference #6M0J (21)) using the software Chimera v1.1 2 (22).

### COVID-19 *in vitro* ACE2 RBD binding assay

The V-PLEX SARS-CoV-2 Panel 22 (ACE2) Kit (Meso Scale Diagnostics, K15562U-2) was used to analyze SARS-CoV-2 neutralizing antibodies in serum from challenged-mice. MSD plates were prepared according to the manufacturer’s protocol and measured on the MSD QuickPlex SQ120. The 10 spots contained (1): RBD B.1.1.529 (2) RBD B.1.351 (3) BSA (4) RBD P.1 (5) BSA (6) RBD B.1.1.7 (7) BSA (8) BSA (9) RBD B.1.617.2 and (10) CoV2 RBD. Serum from each mouse was serially diluted 4 ways, 1:5, 1:50, 1:500 and 1:5,000 and analyzed on the MSD *in vitro* neutralization assay plate. Area Under the Curve analysis of the electrochemiluminescence results was performed using GraphPad Prism.

### Detection of EpF(ab’)_2_ serological levels in mice

To examine the *in vivo* kinetics of EpF(ab’)_2_ and HCP clearance from peripheral circulation, 18-week-old female C57BL/6J mice (Charles River) were intraperitoneally administered 200uL of: 1) Endotoxin Free PBS, 2)10μg EpF(ab’)_2,_ 3)100μg EpF(ab’)_2_, or 4)500μL of HCP, once daily for three consecutive days (0, 1, and 2). Serum was collected for serological analysis of circulating equine IgG and human IgG *via* submandibular bleed on days 1, 2, 3 and 11. ELISAs were performed to assess the persistence of EpF(ab’)2 in the serum post-administration using a modified protocol established previously (23). SpyTag RBD Wu (obtained from Serum Institute of India and Massachusetts Institute of Technology) was used to coat the wells of high binding plates (Pierce, 15041) overnight at 4°C at a concentration of 2μg/mL. The plates were washed three times with PBS-0.1%Tween20 3 times then blocked with 3% non-fat milk in PBS-0.1% Tween 20 for 1 hour shaking at room temperature. After blocking, plates were again washed with PBS-0.1%Tween20 3 times. Serum from individual mice were diluted 1:20 in the first wells of a plate using 1% non-fat milk in PBS-0.1% Tween 20 then serially diluted 1:2 across two plates into 1% non-fat milk in PBS-0.1% Tween 20 (total of 15 dilutions). After 1hr shaking incubation at room temperature, the plates were washed 4 times before adding goat anti-Equine IgG HRP secondary antibody (Novus Biosolutions, NB7421) at a 1:10,000 dilution to all wells for a 1hr incubation shaking at room temperature. Plates were washed 5 more times with PBS-0.1%Tween20 and developed and analyzed as described above.

### Detection of anti-RBD IgG antibodies in HCP in passively immunized mice

Mice were passively immunized with HCP as described above. To measure anti-RBD human IgG levels in mouse serum after HCP administration, ELISAs were performed using the method described above. High binding plates were coated with SpyTag RBD Wu and blocked as previously mentioned. Mouse serum was added at a 1:5 dilution to the first row then serially diluted 1:5 down two plates, discarding before the final row. After 10min shaking incubation at room temperature, plates were washed 4 times and goat anti-human-IgG HRP (Invitrogen, 31410) was added as secondary antibody at a 1:5,000 dilution in 1% non-fat milk in PBS-0.1% Tween 20 and incubated again for 10min. The reactions were developed and ended following the same methods described above. Area Under the Curve calculations were performed to measure titers.

### Cultivation of viral SARS-CoV-2 strains andK18-hACE2 mouse challenge

Alpha and Beta SARS-CoV-2 strains were originally obtained from BEI: hCoV19/England/204820464/2020 (Alpha; NR-54000)(GISAID: EPI_ISL_683466), and hCoV19/South Africa/KRISP-EC-K005321/2020 (Beta; BEI NR-54008) (GISAID: EPI_ISL_678570). The Delta SARS-CoV-2 challenge strain (B.1.617.2 hCoV-19/USA/WV-WVU-WV118685/2021) was first obtained from a patient swab stored in viral transport medium at WVU (GISAID Accession ID: EPI_ISL_1742834). After primary acquisition and sequencing, all SARS-CoV-2 variant strains were propagated in Vero E6 cells (ATCC-CRL-1586) to prepare challenge doses from the first (Alpha, Delta) or second (Beta) passage. Female 12-13-week-old K18-hACE2 mice were IP anesthetized with 80mg/kg ketamine (Patterson Veterinary 07-803-6637)/xylazine (Patterson Veterinary 07-808-1947) then intranasally challenged with 25μL per nostril (50μL total) of 10^3^ PFU SARS-CoV-2 liquid stock.

### Disease scoring of SARS-CoV-2 challenged mice

SARS-CoV-2 challenged K18-hACE2 mice were evaluated daily starting on the day of challenge using in-person health assessments as well as the SwifTAG Systems video monitoring system for 11 days. During the health assessments, body weight and rectal temperature were measured and mice were scored for their appearance and behavior using a previously described scoring system (23). Scores for weight loss (scale 0-5 up to 20% weight loss), appearance (scale 0-2), activity (scale 0-3), eye closure (scale 0-2), and abnormal respiration (scale 0-2) were given on a scale such that 0 represented normal mouse behavior and appearance, and the highest number represented the most severe phenotype. Each mouse’s overall score on each day was recorded as the sum of each category. Mice were euthanized prior to the end of the experiment if their disease score reached a 5 or above, or they experienced 20% weight loss consistent with morbidity. Each experimental group’s cumulative disease score was reported as the total of scores from each mouse in the group on that day. If mice in the group were euthanized before day 11, their score on the day of euthanasia was used for calculation of each subsequent day’s cumulative score.

### Euthanasia and tissue collection

All mice were euthanized on day 11 if they had not received a daily disease score of 5 resulting in earlier euthanasia. Euthanasia was performed using an IP injection of Euthasol (390mg/kg) (Pentobarbital) followed by cardiac puncture as a secondary measure of euthanasia. Cardiac puncture blood was collected (BD Microtainer gold serum separator tubes, 365967) and centrifuged (15,000 x *g*, 5 minutes) to separate the serum. Nasal wash of each mouse was collected by pushing PBS (1mL) by catheter through the nasal pharynx (1mL) into a microcentrifuge tube. To prepare for downstream analysis and to inactivate virus, 500μL of nasal wash was added to 1mL of TRI reagent for RNA purification and the remainder of the nasal wash was treated with 1% triton by volume for serological analysis. Lungs were dissected and separated into right and left lobes: left lobes were saved in 10% neutral buffered formalin for histopathology; right lobes were homogenized in 1mL of PBS using gentleMACS C tubes (Miltenyi Biotec, 130-096-334) on the m_lung_02 program utilizing the gentleMACS Dissociator. From the homogenate, 300μL was added to 1mL of TRI Reagent (Zymo research) for RNA purification and 300μL was centrifuged at 15,000 x *g* for 5 minutes to separate the supernatant for downstream analyses. In a similar manner, brains were collected and homogenized in 1mL PBS and 500μL of the homogenate was added to 1000μL of TRI Reagent for RNA purification.

### qPCR SARS-CoV-2 viral copy number analysis of lung, brain, and nasal wash

RNA was purified from nasal wash, lung, and brain homogenates following the manufacturer’s protocol for the Direct-zol RNA miniprep kit (Zymo Research, R2053). qPCR using the Applied Biosystems TaqMan RNA to CT One Step Kit (Ref: 4392938) was performed on all samples to measure viral copy number in lung, nasal wash, and brain with specifications for each reaction that were described previously (23).

### Histological analysis of lung tissue

The left lobes of the lung from each mouse were fixed in 10mL of 10% neutral buffered formalin. Fixed lungs were sent to the WVU Pathology Core Facilities where they were paraffin embedded, sectioned at 5μm, and stained with hematoxylin-eosin (H&E). Slides of H&E-stained lung sections were sent to iHisto where they were blindly evaluated by Christopher Gibson V.M.D., Ph.D., DACVP. Tissue sections were scored for chronic and acute inflammation within the lung parenchyma, surrounding blood vessels, and airways. Scores were based on standard scoring criteria: 0 – none, 1 – minimal, 2 – mild, 3 – moderate, 4 – marked, 5 – severe. Chronic inflammation was characterized by mononuclear infiltrates composed of lymphocytes and plasma cells. Scoring of chronic inflammation was performed separately for the lung parenchyma, blood vessels, and airways (bronchi and bronchioles). Infiltrating inflammatory cell subsets (lymphocytes vs histiocytes) were scored separately as they are not usually seen concurrently.

### Statistical analyses

All murine passive immunization and challenge experiments were performed with an n=5 mice/group. Antibody peptide binding assays, *in vitro* neutralization assays, and ELISA detection in serum were performed for each individual sample with a minimum of two technical replicates. qPCR viral RNA detection was performed in triplicate. Detection of EpF(ab’)_2_ in the serum during kinetic studies was performed using a one-sample *t*-test comparison to the value of 0 (AUC for naïve animals). Differences in survival between each treatment group and the PBS control group were determined using a log-rank Mantel-Cox test. Simple linear regressions were performed to determine correlations between temperature and weight loss during infection. Finally, ordinary one-way ANOVA with Dunnett’s multiple comparisons test was used for comparison of multiple data sets following a normal distribution and Kruskal-Wallis with Dunn’s multiple comparisons test for non-parametric distributed datasets.

### Biosafety, animal, and ethics statement

K18-hACE2-mouse (B6.Cg-Tg(K18-ACE2)2Prlmn/J; JAX strain number #034860) challenge studies were performed under West Virginia University IACUC protocol number 2009036460. Viral propagation and challenge studies utilizing SARS-CoV-2 occurred in Biosafety Level 3 (BSL-3) facilities at West Virginia University (IBC 20-04-01). Tissues and biological samples that contained SARS-CoV-2 were treated to inactivate the virus before they were moved from BSL-3 to BSL-2 laboratory space as follows: serum, supernatants, and residual tissue homogenates were treated with 1% Triton per volume; full tissues for histology were fixed in 10% neutral buffered formalin; tissue homogenates for RNA analysis were added to TRIzol (Zymo research R2050-1) reagent at 1:2 or greater ratio by volume.

## Results

### EpF(ab’)_2_ bind and neutralize SARS-CoV-2 variants

The overall objective of this study was to evaluate the protective efficacy of EpF(ab’)_2_ administration during challenge with different SARS-CoV-2 VOC in mice to determine their potential use as a therapeutic treatment. To do this, EpF(ab’)_2_ were produced by hyper-immunizing horses with a yeast-purified RBD and HBsAg conjugate. The RBD sequence of the WA-1 ancestral SARS-CoV-2 strain was selected for this work, and RBD were conjugated to HBsAg *via* Spycatcher technology (24). Horses were immunized with a total of 6 doses delivered intramuscularly every 1 to 2 weeks over the course of 48 days. Titers and antibody neutralizing activity were measured over time. At time of collection, antibodies had reached a titer >1,024,000 and a plaque reduction *in vitro* neutralization titer 50 (PRNT50) of 2,621,440 (data not shown).

**Figure 1:**
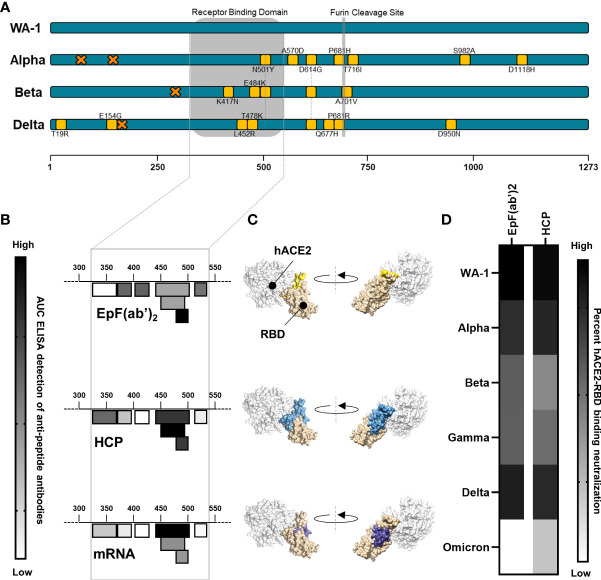
EpF(ab’)_2_binds to RBD and neutralize RBD-hACE2 interaction. **(A)** Graphical representation of the mutations on each of the viral strains used in this study. Amino-acid deletions are depicted with orange crosses and point mutations with yellow squares. **(B)** Heat map of the ACU from ELISA detection of the binding of EpF(ab’)_2_, HCP, and mRNA sera to seven peptides in the RBD region of the SARS-CoV-2 spike protein. Darker colors represent higher binding to the peptides. **(C)** 3D representation of the SARS-CoV-2 RBD binding to the hACE2 protein. Areas of highest binding as determined in B are highlighted in colors for EpF(ab’)_2_ (yellow), HCP (blue), and mRNA (purple). **(D)** Heat map representing percent inhibition of RBD binding to hACE2 determined using neutralization assays at non-saturating concentrations of antibody for EpF(ab’)_2_ and HCP. Darker colors represent higher binding neutralization.

The binding interactions between EpF(ab’)_2_ and RBD were first characterized by performing ELISA assays against seven peptides spanning the RBD region of the SARS-CoV-2 Spike protein (**Figures 1A**, **B**). The sequence of these peptides was selected based on both predictions of the linear B cell epitopes within the RBD region as well as preidentified B cell epitopes. Binding of EpF(ab’)_2_ to these peptides was compared to the binding of antibodies found in the serum of human convalescent plasma (HCP) or in serum from Pfizer mRNA vaccine immunized (3µg) mice in order to measure the relative protective efficacy of the therapeutic antibody fragments to antibodies raised by vaccination or natural infection. The HCP sample was obtained during the earliest phase of the pandemic (May 2020) from an infected patient. ELISA RBD peptide binding assays showed that EpF(ab’)_2_ binds to 6 out of 7 of the RBD peptides and that binding was highest on residues 480-499 (**Figure 1B** and **Table S1**). Antibodies present in HCP also bound to 6 out of 7 peptides tested, with the highest binding on residues 440-501(**Figure 1B** and **Table S1**). The binding spectrum of the serum from Pfizer mRNA vaccinated mice was the narrowest, with binding to 4 out of 7 peptides, and highest binding was observed for residues 440-501 (**Figure 1B** and **Table S1**). Interestingly, for EpF(ab’)_2_, HCP, and mRNA vaccination, highest antibody binding was observed at the residues located directly at the site of binding between RBD and hACE2 (**Figure 1C**).

To determine if antibody binding was capable of inhibiting the interaction between RBD and the hACE2 receptor, *in vitro* ACE2-RBD binding assays were performed using RBD from the ancestral strain (e.g. WA-1), Alpha, Beta, Gamma, Delta, and Omicron variants. EpF(ab’)_2_ best blocked the binding of hACE2 to RBD from the ancestral strain (IC50 = 2.283 μg/ml), followed by Delta (IC50 = 3.553 μg/ml), Alpha (IC50 = 4.337 μg/ml), Beta (IC50 = 7.245 μg/ml), and Gamma (IC50 = 7.377 μg/ml). Approximately 30-fold more EpF(ab’)_2_ was required to block the binding of Omicron (BA.1) RBD to hACE2 compared to RBD from the ancestral SARS-CoV-2 strain. Percent *in vitro* neutralization of these antibodies, indicated by blocked ACE2-RBD binding, at a non-saturating dose (0.01 mg/ml) is represented in **Figure 1D**. A similar *in vitro* neutralization profile was observed for HCP, with the highest binding occurring against RBD from the ancestral strain, followed by Alpha. Interestingly, approximately 3.5- to 7.3-fold more antibody was required to reach the neutralization IC50 for Beta, Gamma, and Delta. Approximately 19.3-fold more antibody from HCP was also required to neutralize 50% of the hACE2 binding of Omicron RBD. Overall, the data indicate that hyperimmunization of horses with an RBD HBsAg conjugate followed by enzymatic treatment to generate EpF(ab’)_2_ leads to the production of antibodies that bind the region of RBD involved in hACE2 interactions and are capable of neutralizing binding to the receptor of most SARS-Cov-2 VOC.

### Passive immunization with EpF(ab’)_2_ is associated with the presence of circulating EpF(ab’)_2_ that decline over time

**Figure 2:**
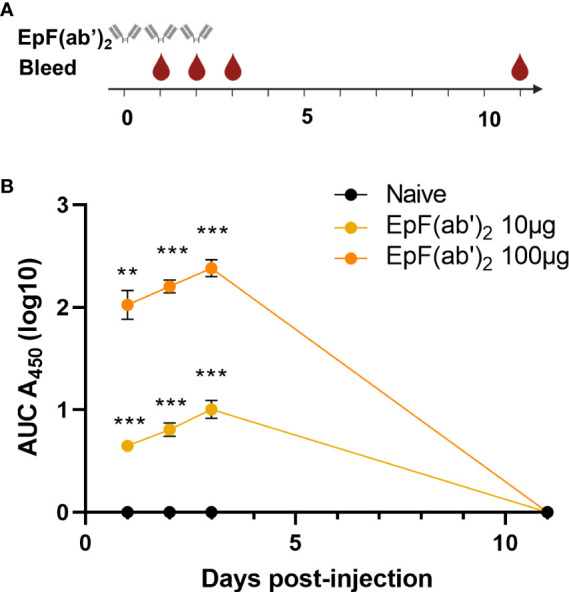
Kinetics of EpF(ab’)_2_ in serum after passive immunization in C57BL/6J. **(A)** Graphical representation of the timeline for EpF(ab’)_2_ administration and bleeding. The timeline represents days. **(B)** AUC representation of EpF(ab’)_2_ ELISA detection in the serum at days 1, 2, 3, and 11 in C57BL/6J mice passively immunized with 10 or 100μg of EpF(ab’)_2_. Statistical analyses were performed as one-sample *t*-tests compared to the naïve animals (**p<0.01; ***p<0.001).

The primary objective of this work was to determine if EpF(ab’)_2_ can be used for the treatment of SARS-CoV-2 infection through passive immunization. To this extent, we first evaluated the kinetics of EpF(ab’)_2_ in sera of mice at various timepoints following intraperitoneal administration. The therapeutic doses tested were selected based on existing experimental data in K18-hACE2 mice available when this kinetics study was performed. Rosenfeld et al. achieved partial (40%) protection in a lethal model of SARS-CoV-2 infection in K18-hACE2 mice utilizing 10μg of a single-chain human-Fc recombinant antibody, and achieved full protection with a 100μg dose (25). These doses correspond to approximately 7.3mg (10μg dose) and 73mg (100μg dose) of EpF(ab’)_2_ per adult human with an average weight of 65kg. To determine the kinetics of both 10μg and 100μg doses in mice, we used C57BL/6J mice, the parental strain of K18-hACE2-transgenic mice. EpF(ab’)_2_ were administered interperitoneally on days 0, 1, 2 of the experiment and anti-Spytag RBD antibody titers were determined at day 1, 2, 3 and 11 (**Figure 2A**). We observed that EpF(ab’)_2_ were detectable and significantly higher than the naïve animals at day 1 post-primary administration (**Figure 2B**). The titers continued to increase out to day 4 for both treatment groups but were cleared to levels below limits of detection by day 11. These data indicate that high levels of circulating EpF(ab’)_2_ can be achieved in mice by passive immunization and remain high for a short period of time post initial administration.

### Treatment with EpF(ab’)_2_ helps reduce disease scores and improves survival in K18-hACE2 challenged mice

**Figure 3:**
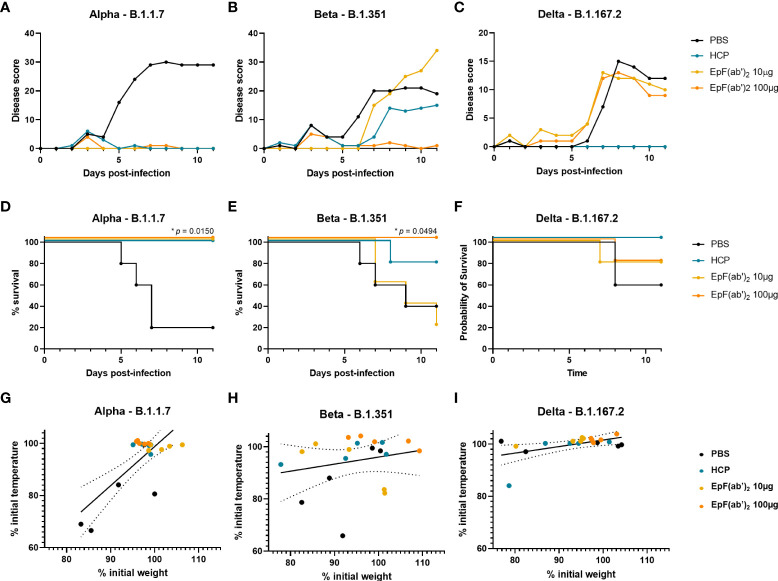
Treatment with EpF(ab’)_2_ helps reduce disease scores and improves survival in K18-hACE2 challenged mice. Cumulative disease scores of K18-hACE2 mice infected with Alpha **(A)**, Beta **(B)**, and Delta **(C)** over time. Kaplan Meyer representation of the percentage of survival over time of mice infected with Alpha **(D)**, Beta **(E)**, and Delta **(F)**. Correlations between weight loss, represented as percentage of weight at day 0, and temperature loss, represented as percentage of temperature at day 0 of K18-hACE2 mice infected with Alpha **(G)**, Beta **(H)**, and Delta **(I)**. Differences in survival between each treatment group and the PBS control group were determined using a log-rank Mantel-Cox test (*p<0.05). Correlations were performed by fitting the data to a simple linear regression.

To determine the therapeutic potential of EpF(ab’)_2_ against lethal SARS-CoV-2 infection *in vivo*, K18-hACE2 mice were challenged with 10^3^ PFU of Alpha, Beta, or Delta SARS-CoV-2 strains. Immediately following challenge (day 0), mice were intraperitoneally administered either 10 or 100μg of EpF(ab’)_2_, 500μL HCP, or 500μL PBS as vehicle control. EpF(ab’)_2_ as well as HCP and PBS administration were repeated on day 1 and day 2 post-challenge, as described in **Figure 2A**. Scorable disease phenotypes began to appear around day 5-6 for all three variant challenge groups and continued to worsen over days 8 to 11 (**Figures 3A**–**C**). Disease manifestations included reduced activity, weight loss, changes in grooming activity, hunched posture, eye closure, and visible alterations in normal respiration. Disease was most severe in mice challenged with Alpha (80% death), followed by Beta (60% death) and Delta (40% death) in the PBS control group (**Figures 3A**–**C**). We observed correlations between weight and temperature loss at the point which all mice challenged with Alpha reached morbidity (**Figure 3G**; R^2^ = 0.5673). Weight loss was comparatively less severe in PBS treated mice challenged with Beta or Delta (**Figures 3H**, **I**; R^2^ = 0.05014 and 0.2728 respectively; **Figures S1**, **S2**). In mice challenged with Alpha, both doses of EpF(ab’)_2_ and HCP significantly protected mice against mortality (**Figure 3D**) and prevented any increase in disease scores (**Figure 3A**). In mice challenged with Beta, morbidity and increased disease scores were prevented by administration of 100μg EpF(ab’)_2_ (**Figures 3B**, **E**). The effect of HCP was intermediate, and 10μg EpF(ab’)_2_ had no effect on disease scores or survival outcomes. Delta-challenged animals showed overall lower disease scores (**Figure 3C**) than those challenged with Alpha or Beta, and the % survival observed in the PBS group was higher than Alpha at the same dose. For Delta groups, only HCP was able to prevent an increase in disease scores (**Figure 3C**) and protected all mice against mortality (**Figure 3F**). Although not significantly different, administration of either dose of EpF(ab’)_2_ had an intermediate effect on Delta challenged mice survival (**Figure 3F**). Overall, these data show that disease progression is variable between each variant and that HCP and 100μg EpF(ab’)_2_ have the highest impact in reducing disease manifestation and mortality in K18-hACE2 mice.

**Figure 4:**
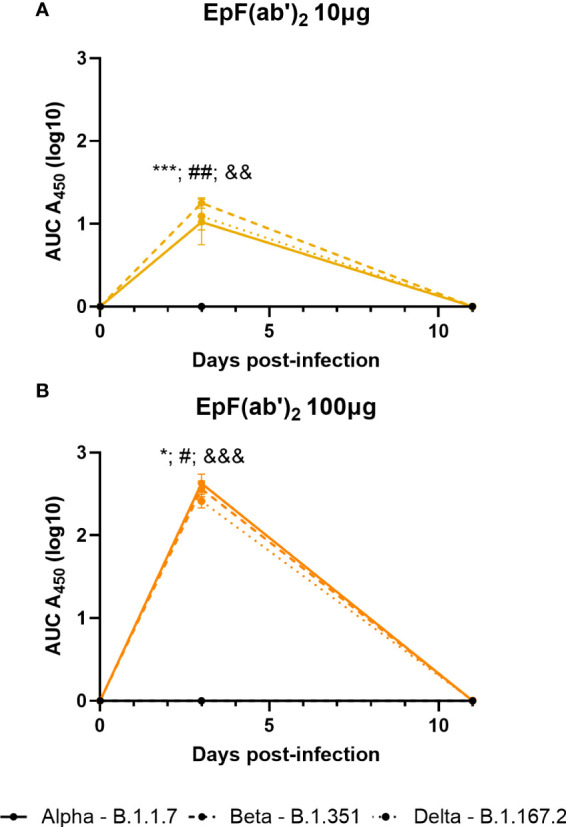
Kinetics of EpF(ab’)_2_ in serum after passive immunization in challenged K18-hACE2 mice. AUC representation of EpF(ab’)_2_ ELISA detection in the serum at days 3 and 11 in K18-hACE2 mice challenged with Alpha, Beta, and Delta, and passively immunized with either 10μg **(A)** or 100μg **(B)** of EpF(ab’)_2_. Statistical analyses were performed as one-sample *t*-tests compared to the naïve animals (*p<0.05; ***p<0.001; ^#^p<0.05; ^##^p<0.01; ^&&^p<0.01; and ^&&&^p<0.001). Results from statistical analyses of comparisons of Alpha to naïve animals are represented with *, Beta to naïve animals with #, and Delta to naïve animals with &.

Similar to as observed in C57BL/6J mice (**Figure 2B**), passive immunization of K18-hACE2 mice led to high levels of circulating anti-Spytag RBD EpF(ab’)_2_ titers at day 3 post-challenge (**Figure 4**). Circulating antibody levels were dose-dependent and declined to non-detectable levels by day 11. In contrast, HCP levels were detectable in mice at both days 3 and 11 (data not shown). Challenge with various SARS-CoV-2 strains did not alter the concentration of circulating EpF(ab’)_2_ and HCP in challenged mice (**Figure 4**).

### Passive immunization with EpF(ab’)_2_ and HCP decreases viral burden in SARS-CoV-2 challenged K18-hACE2 mice

**Figure 5:**
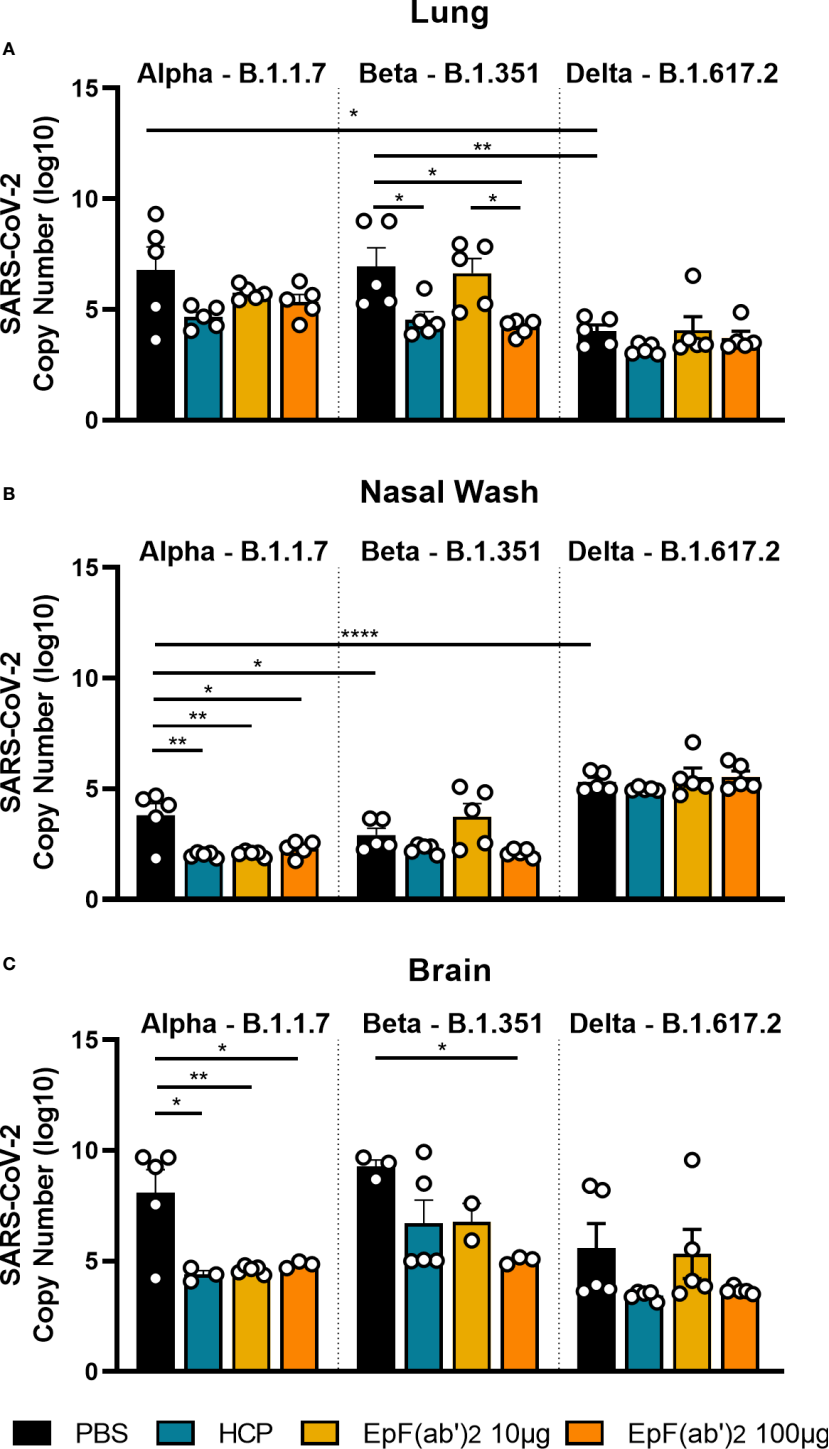
Viral burden in lung, nasal wash and brain detected by qPCR. qPCR detection of SARS-CoV-2 viral burden in the lung **(A)**, nasal wash **(B)**, and brain **(C)** at time of euthanasia in mice infected with Alpha, Beta, or Delta. Kruskal-Wallis with Dunn’s multiple comparisons test was used to compare viral burden in the tissue (*p<0.05; **p<0.01; ****p<0.0001).

During viral infection, antibodies that bind virions participate in neutralization, blocking of viral entry and replication, and promote virion clearance. So far, we have shown that EpF(ab’)_2_ can block RBD binding to hACE2 (**Figure 1**) and help decrease disease scores and death following challenge with SARS-CoV-2 (**Figure 2**). To determine if EpF(ab’)_2_ can also aid in decreasing viral burden and prevent viral replication, we performed qRT-PCR on lung, nasal wash, and brain tissue from mice challenged with Alpha, Beta, or Delta SARS-CoV-2 variants collected at morbidity or the terminal point of the study. Viral RNA copy numbers were calculated using amplification of the nucleocapsid gene and a standard curve with known viral RNA copy numbers. We first observed that Delta had lower viral burden in the lung than Alpha or Beta in naïve animals, but higher burden in the nasal wash (**Figure 5**), suggesting a distinct distribution of each variant within the respiratory tract. Consistent with the decreased disease scores and increased survival measured in **Figure 2**, we observed that HCP and both doses of EpF(ab’)_2_ were able to decrease the viral RNA burden in the nasal wash and brain of mice challenged with the Alpha variant (**Figures 5B**, **C**). Also consistent with disease outcome, only the HCP and the 100μg dose of EpF(ab’)_2_ were able to significantly decrease the viral RNA loads in the lung of mice infected with the Beta variant. The highest dose of EpF(ab’)_2_ was also able to significantly decrease the viral RNA burden in the brain of mice challenged with the Beta variant (**Figure 5C**). Regardless of the tissue studied, no significant differences in viral RNA burden were observed between any of the treatment groups in mice challenged with the Delta variant (**Figure 5**). Overall, this data supports that passive immunization with 100μg EpF(ab’)_2_ and HCP protect mice from severe disease after challenge with the Alpha and Beta SARS-CoV-2 variants by reducing viral burden in the lung and the brain.

### Treatment with EpF(ab’)_2_ does not ameliorate chronic lung inflammation in SARS-CoV-2 infected mice

**Figure 6:**
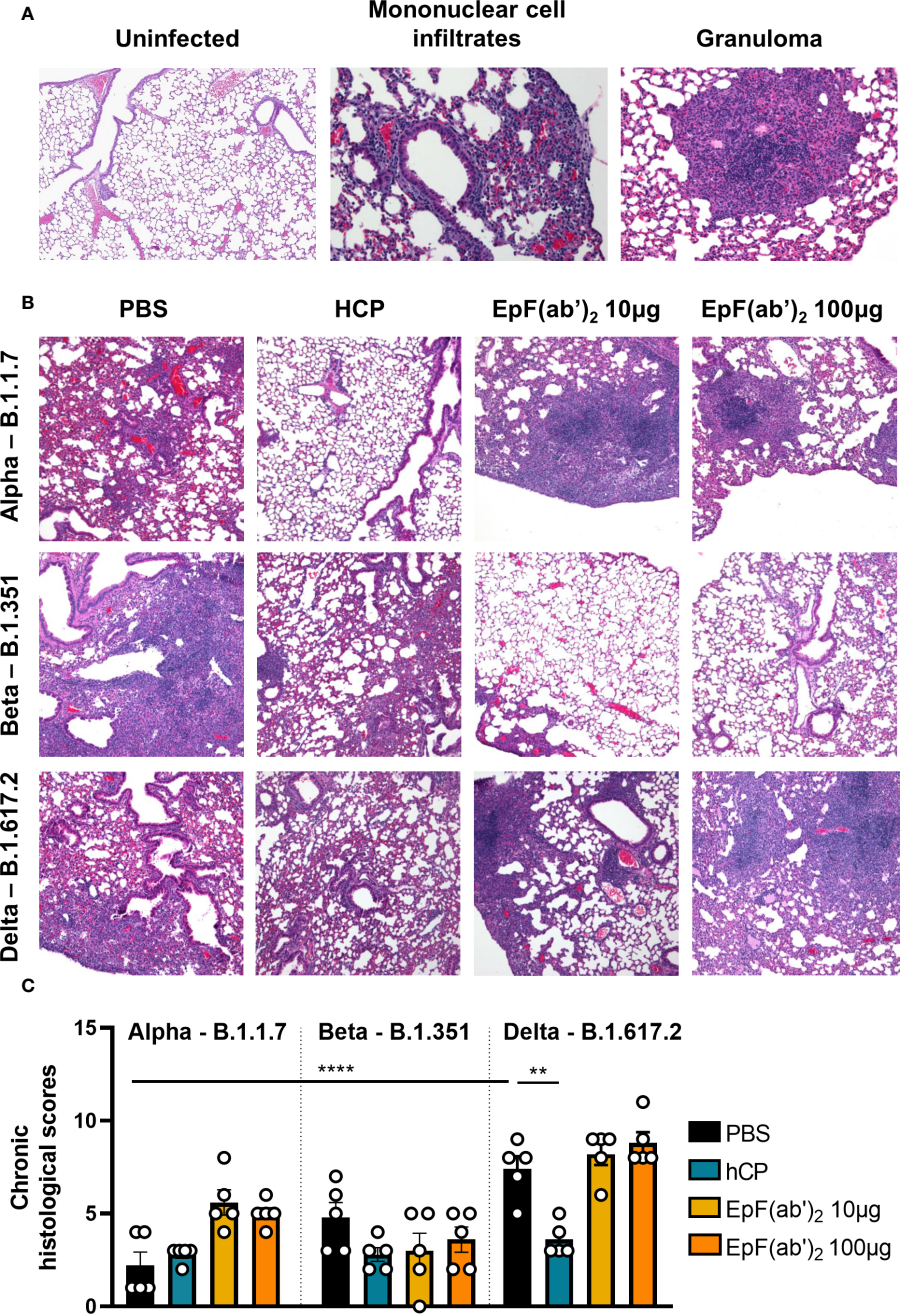
SARS-CoV-2 causes chronic inflammation in the lung of infected K18-hACE2 mice. **(A)** Histopathological analysis of hematoxylin-eosin-stained sections of lung from non-infected and SARS-CoV-2 infected K18-hACE2 mice (Representative images at 200x magnification). Left: Characteristic features of a non-infected lung (left); Center: detection of increased numbers of mononuclear cells within the parenchyma (asterisk), surrounding blood vessels (arrowhead), and surrounding bronchi/bronchioles (arrow) in the lung of mice infected with B.1.1.7; Right: Detection of granulomatous inflammation in the lung of mice infected with B.1.351. **(B)** Histopathological analysis of hematoxylin-eosin stained sections of lung from SARS-CoV-2 infected K18-hACE2 mice untreated, or treated with HCP or EpF(ab’)_2_ (representative images at 100x magnification). **(C)** Chronic histological scores from hematoxylin-eosin stained sections of lung from SARS-CoV-2 infected K18-hACE2 mice untreated, or treated with HCP or EpF(ab’)_2_. \Differences between infected groups are denoted with brackets (One-way ANOVA with Tukey’s multiple comparison test). **p < 0.01, and ****p < 0.0001.

To determine the effect of SARS-CoV-2 challenge and EpF(ab’)_2_ treatment on lung pathology, histopathological scoring of acute and chronic inflammation was performed. Lung infection from Alpha was characterized by chronic perivascular inflammation with mononuclear infiltrates composed of lymphocytes and plasma cells (**Figure 6A**; average total inflammation score of 2.8). Although not statistically significant, treatment with HCP and EpF(ab’)_2_ (10 and 100μg doses) was associated with greater parenchymal and peribronchiolar inflammation than the non-treated challenged group in mice infected with B.1.1.7 (**Figures 6B**, **C**). Lung infection from Beta was more severe with an average total inflammation score of 5.4 and presented extensive multifocal granulomatous inflammation not identified from Alpha (**Figure 6A**). In mice infected with Beta, HCP and EpF(ab’)_2_ (10 and 100μg doses) treatments were associated with milder peribronchiolar and parenchymal inflammation, and lower granulomatous inflammation than the non-treated infected group, although these changes were not statistically significant (**Figures 6B**, **C**). Surprisingly, Delta triggered the highest levels of chronic lung inflammation compared to Alpha and Beta with an average total inflammation score of 9.4 (**Figures 6B**, **C**). Inflammation was multifocal and evenly distributed between the lung parenchyma, airways, and surrounding blood vessels. Only HCP was able to significantly reduce the chronic inflammation caused by Delta. Similar pathology to that observed in the PBS-treated Delta group was also observed in EpF(ab’)_2_ treated animals challenged with this variant (average total inflammation score of 11 for the 10μg treatment group and 10.6 for the 100μg treatment group). Overall, chronic inflammation (marked by infiltration of lymphocytes, plasma cells and macrophages) was the predominant form of inflammation in this study, regardless of the viral strain. Histopathological observations suggest that treatment with EpF(ab’)_2_ did not improve the chronic inflammation scores in animals infected with any of the three variants tested. These findings highlight the importance of conducting in-depth characterization of the effects of experimental therapeutics such as EpF(ab’)_2_ and HCP on additional parameters outside of disease severity and death.

## Discussion

In this study, we evaluated the therapeutic use of SII’s EpF(ab’)_2_ (raised in response to vaccination with RBD conjugated to HBsAg) against SARS-CoV-2 infection in a pre-clinical model of passive immunization and challenge. A similar polyclonal antibody fragment specific to RBD was previously utilized in Argentina under Emergency Use Authorization that proved to be clinically effective for supporting the clearance of SARS-CoV-2 infection (26). We demonstrated that EpF(ab’)_2_ binds RBD and blocks binding of the RBD of most variants tested to the hACE2 receptor. We also demonstrated that the 100μg dose has the highest therapeutic effect by decreasing disease scores and viral loads in infected tissues, and by increasing murine survival.

The work presented here provides an additional proof of concept for the use of EpF(ab’)_2_ antibodies for therapeutic use against infectious diseases (26–32). Previous work from our laboratory has shown that RBD conjugated with HBsAg can also be used as an effective vaccine antigen for the protection of K18-hACE2 mice against SARS-CoV-2 infection (23). While the whole spike protein is more highly immunogenic and contains more B and T cell epitopes (33, 34), RBD is more soluble and can be produced more easily and at lower costs. Vaccination with RBD conjugated with HBsAg allowed for the generation of antibodies that target the binding site of RBD to the hACE2 receptor, allowing for *in vitro* blocking of RBD-hACE2 binding. Despite the fact that the RBD antigen was designed after the ancestral strain of SARS-CoV-2, we observed *in vitro* blocking of the binding of Alpha, Beta, Gamma, and Delta SARS-CoV-2 RBD variants to the hACE2 receptor. We additionally confirmed that EpF(ab’)2 can neutralize the Alpha variant in an authentic viral neutralization assay using Vero E6 cells (data not shown), which correlated to what was seen in the binding assay. Limited *in vitro* neutralization was observed with the Omicron variant which was unsurprising after clinical reports of reduced *in vitro* neutralization ability of vaccine-raised and convalescent antibodies against the variant (35–39).

Passive immunization with 100μg of EpF(ab’)_2_ provided the highest protection against clinical manifestation of the disease, viral burdens, and death against challenge with Alpha and Beta SARS-CoV-2 variants. Compared to HCP treatment, this dose of EpF(ab’)_2_ also performed equally well or better at reducing disease scores, and minimizing viral tissue burden from Alpha and Beta. This observations supports the superiority of highly purified, polyclonal antibodies as therapeutics compared to non-specific convalescent plasma which may have undesirable side effects (40). Importantly, this dosage (100μg) can easily be administered in humans. The 10μg dose of EpF(ab’)_2_ was capable of protecting against challenge with the Alpha variant. We speculate that the limited efficacy of the 10μg dose against the other variants is due, in part, to the relatively quick clearance of the EpF(ab’)_2_ from the blood after day 3. EpF(ab’)_2_ have a relatively short plasma half-life due to both their small size and their inability to bind the FcRn receptor (41). This also gives the therapeutic an advantage of easy clearance from system after effectively neutralizing the viral load. In this study, EpF(ab’)_2_ treatment was administered on the same day as intranasal viral challenge and subsequently on days 1 and 2 post-challenge. Since mice start to display clinical symptoms and begin to succumb to infection after day 5, it is conceivable that administration of EpF(ab’)_2_ needs to be extended past day 2 post-challenge. Future studies evaluating the efficacy of sustained dosage of EpF(ab’)_2_ as well as the potential prophylactic use of these antibodies for treatment of SARS-CoV-2 infection should be considered.

Efficacy data of EpF(ab’)_2_ treatment in mice challenged with the Delta variant was inconclusive. This is likely due in part to the fact that infection with the Delta variant causes different disease manifestations and progresses uniquely compared to challenge with the Alpha and Beta variants in preclinical models (42–44). First, while 10^3^ PFU of the Alpha and Beta variants are sufficient to induce over 60-80% death in K18-hACE2 mice, the same dose of the Delta variant only caused death of 40% of the mice. This makes it difficult to observe improvement of disease in this model and suggests that higher doses of the Delta variant should be used for future challenge studies. At the chosen challenge dose of 10^3^ PFU, Delta does not cause the same disease scores as Alpha or Beta (**Figure 3**). One caveat of this study is the fact that we used HCP from a patient infected with ancestral SARS-CoV-2. It is possible that if HCP from Delta infected humans was used, more neutralization and lower disease after challenge could be observed. We also observed that while all variants caused significant weight loss in naïve animals, challenge with the Delta variant did not cause the decrease in body temperature observed with the two other variants. The distribution of the viral loads in the respiratory tract were also different, with higher levels of virus in the nares and lower viral loads in the lung in animals infected with the Delta variant compared to Alpha and Beta. Most strikingly, while infection with Delta caused lower clinical scores and higher survival, it was also associated with the highest levels of chronic inflammation in the lung. Altogether, these data underscore the importance of evaluating therapeutic efficacy against variants of SARS-CoV-2. Unfortunately, we were unable to determine EpF(ab’)_2_ efficacy against Omicron in mice as this strain is not lethal in K18-hACE2 mice, even at high doses such as 10^5^ PFU (45). We propose that as SARS-CoV-2 variants continue to arise, the formulation of the RBD HBsAg vaccine needs to be modified to take in account important changes in the sequence of RBD in these mutants.

We appreciate that this study does have some limitations that would need to be assessed as EpF(ab’)_2_ moves towards potential use in humans. For one, in these experiments, EpF(ab’)_2_ was administered on the day of viral challenge, which would be difficult to recapitulate in human cases of COVID-19. Additional studies would need to investigate administration at later points in the disease timeline to assess the window in which the therapeutic is most effective. Another possible concern for EpF(ab’)_2_ use in humans is the potential reactivity between an antibody-based therapeutic and the host immune system. Antibody dependent enhancement (ADE) is a phenomenon where traditionally vaccine-generated (or in this case therapeutic-based) antibodies enhance viral replication allowing for worsened disease than would’ve occurred without the antibodies. ADE could limit the number of doses of EpF(ab’)_2_ that are usable in humans and would require close monitoring in clinical trials. One final limitation of our study is the lack of assessment of EpF(ab’)_2_ against the Omicron subvariants BA.4 and BA.5 which are rising to dominance in the world as of summer 2022. These variants pose the highest resistance to previously raised (whether by vaccination or prior infection) antibodies yet due to its mutations in the spike protein and RBD, and are likely to reduce the efficacy of EpF(ab’)_2_ which were raised against RBD of the SARS-CoV-2 ancestral strain. To protect against these strains, it may be necessary to raise EpF(ab’)_2_ against newer variants of SARS-CoV-2.

In this study, EpF(ab’)_2_ efficacy data was corroborated using disease scores, survival, and viral burden in the airways and the brain. One caveat of this study is that RT-qPCR only evaluates RNA copy number and cannot distinguish between infective particles and inert genetic material. We did not determine the effect of EpF(ab’)_2_ on viral burden in infected tissue using plaque forming assays, which should be considered in future studies. In addition, the assessment of viral burden does not allow us to differentiate between clearance of the virus, inhibition of viral replication, or prevention of virus dissemination. Additional studies should be considered to determine the exact mechanism of action of EpF(ab’)_2_ in the treatment of SARS-CoV-2 infections. Overall, the pre-clinical data from this study support the efficacy of EpF(ab’)_2_ for the treatment of SARS-CoV-2 infection and warrant the pursuit of clinical studies in patients. This type of therapeutic platform could generate unique opportunities to produce affordable—cost effective and efficient—treatments for viral infection in developing countries.

## Data availability statement

The raw data supporting the conclusions of this article will be made available by the authors, without undue reservation.

## Ethics statement

The animal study was reviewed and approved by West Virginia University Institutional Animal Care and Use Committee.

## Author contributions

The experiments in this study were designed by USS, MSV, FHD and JRB. SII provided EpF(ab’)_2_ reagent for *in vivo* evaluation and RBD-HBsAg conjugate for serological analysis. SARS-CoV-2 peptide structures were modelled by ES-K and were generated by FHD and JK. EpF(ab’)_2_ peptide binding and neutralization assays were performed by JK, TYW, and MC. TYW, OAM, and NAR performed C57BL/6J kinetics studies. Virus for challenge studies was prepared by MTW and IM. FHD, JRB, BPR, HAC, TYW, KSL, and OAM assisted with *in vivo* mouse challenge, EpF(ab’)_2_ and HCP administration, daily health assessments, and necropsy of animals. Serological analysis *via* ELISA was performed by KSL, NAR and OAM. HAC, KSL, and OAM measured tissue viral RNA burden by qPCR. Data analysis was performed by MB, KSL, TYW, and FHD. MB and KSL prepared data for presentation. MSV, SNR, NK, RD, and USS were involved in study conceptualization. All authors contributed to the writing, review, and edits of this manuscript. All authors contributed to the article and approved the submitted version.

## Funding

These projects were funded by Serum Institute of India. The funder was not involved in the study design, collection, analysis, interpretation of data, the writing of this article or the decision to submit it for publication. These projects were performed at the WVU Vaccine Development Center. West Virginia University’s Vaccine Development Center is supported by the Research Challenge Grant no. HEPC.dsr.18.6 from the WV Higher Education Policy Commission’s Division of Science and Research. The authors acknowledge the support for this project that was provided by the West Virginia University Health Sciences Center’s Vaccine Development Center. The VDC and its investigators are supported by the Research Challenge Grant no. HEPC.dsr.18.6 from the WV Higher Education Policy Commission’s Division of Science and Research.

## Acknowledgments

The Serum Institute of India supports the studies detailed in this publication. EpF(ab’)_2_ was formulated and provided by the Serum Institute of India for trials described in this manuscript. We thank Drs. Peter Perrotta and Katy Smoot for their efforts to acquire human convalescent plasma. The MSD QuickPlex SQ120 used for analysis in the WVU Flow Cytometry & Single Cell Core Facility is supported by the Institutional Development Awards (IDeA) from the National Institute of General Medical Sciences of the National Institutes of Health under grant numbers P30GM121322 (TME CoBRE) and P20GM103434 (INBRE). We thank MSD’s Gaurav Deshmukh for his help designing the MSD *in vitro* neutralization assay; Mary Tomago-Chesney from WVU’s Pathology and Histology Core Facility for her work to prepare tissue sections for analysis; iHisto’s Dr. Christopher Gibson for scoring tissue slides; and Dr. James Denvir for sequencing the SARS-CoV-2WA-1, Alpha, Beta and Delta strains that were utilized for mouse challenge studies. BioRender was used to create the supplementary figures featured in this manuscript. Lastly, we owe our tremendous thanks to Drs. Laura Gibson and Clay Marsh at West Virginia University for their support of our lab’s COVID-19 vaccine research efforts.

## Conflict of interest

Authors MV, SR and US were employed by company Serum Institute of India Pvt. Ltd. Author NK was employed by company Isera Biological Pvt. Ltd.

The remaining authors declare that the research was conducted in the absence of any commercial or financial relationships that could be construed as a potential conflict of interest.

## Publisher’s note

All claims expressed in this article are solely those of the authors and do not necessarily represent those of their affiliated organizations, or those of the publisher, the editors and the reviewers. Any product that may be evaluated in this article, or claim that may be made by its manufacturer, is not guaranteed or endorsed by the publisher.
