# Supplementary figures and images for: Passive immunization with equine RBD-specific Fab protects K18-hACE2-mice against Alpha or Beta variants of SARS-CoV-2
Source: Front Immunol. 2022 Aug 15;13:948431. doi: 10.3389/fimmu.2022.948431 (PMC9450042; doi:10.3389/fimmu.2022.948431)

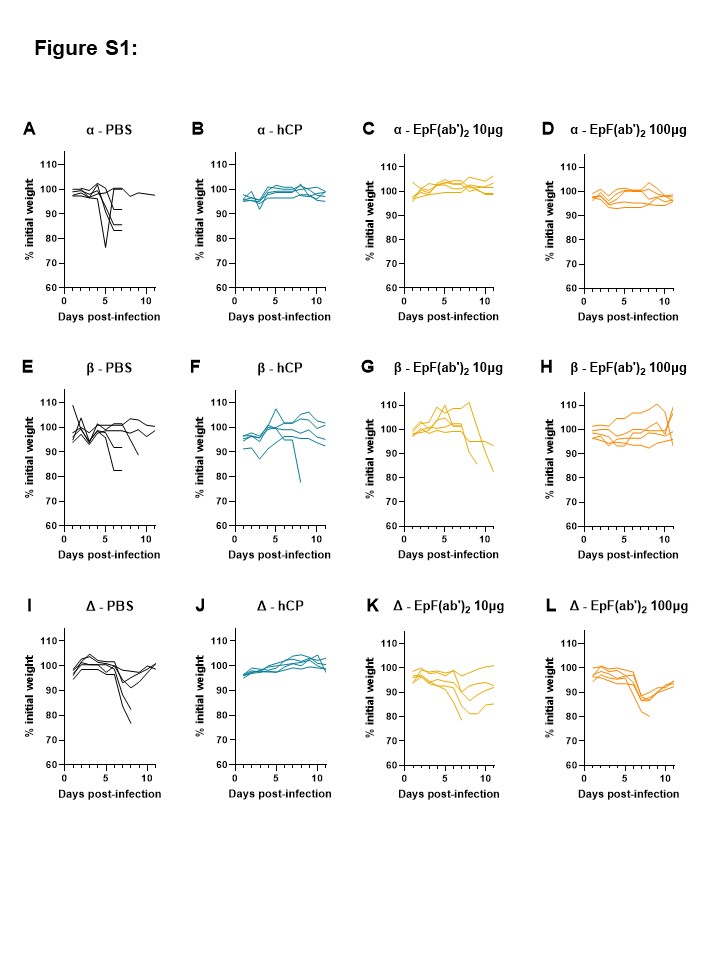

Supplement: Supplementary file 1 [file Image_1.jpeg]

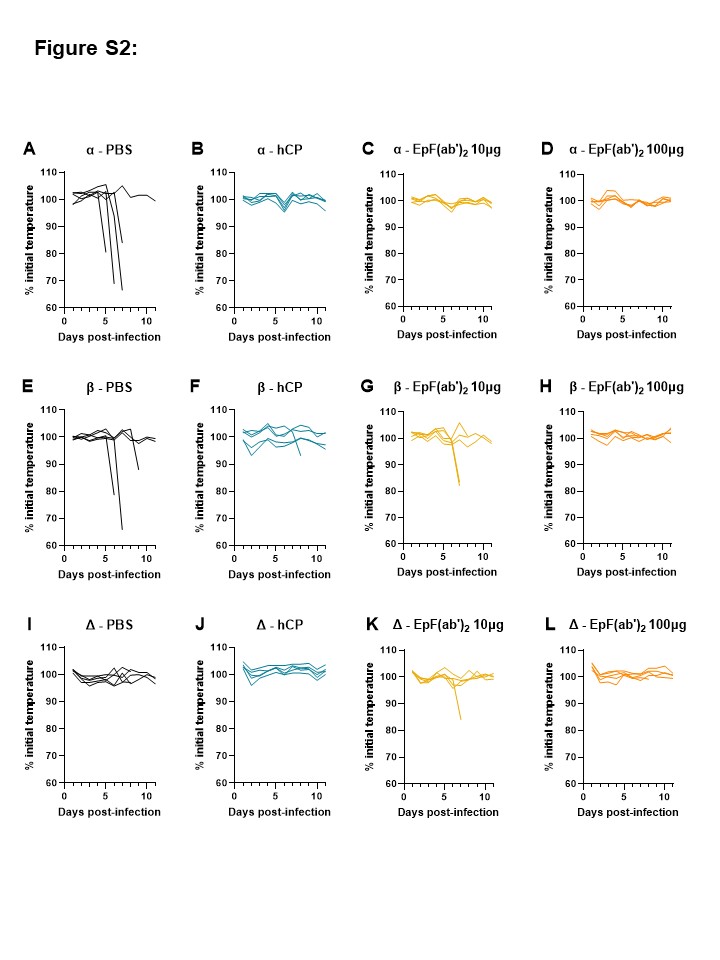

Supplement: Supplementary file 2 [file Image_2.jpeg]

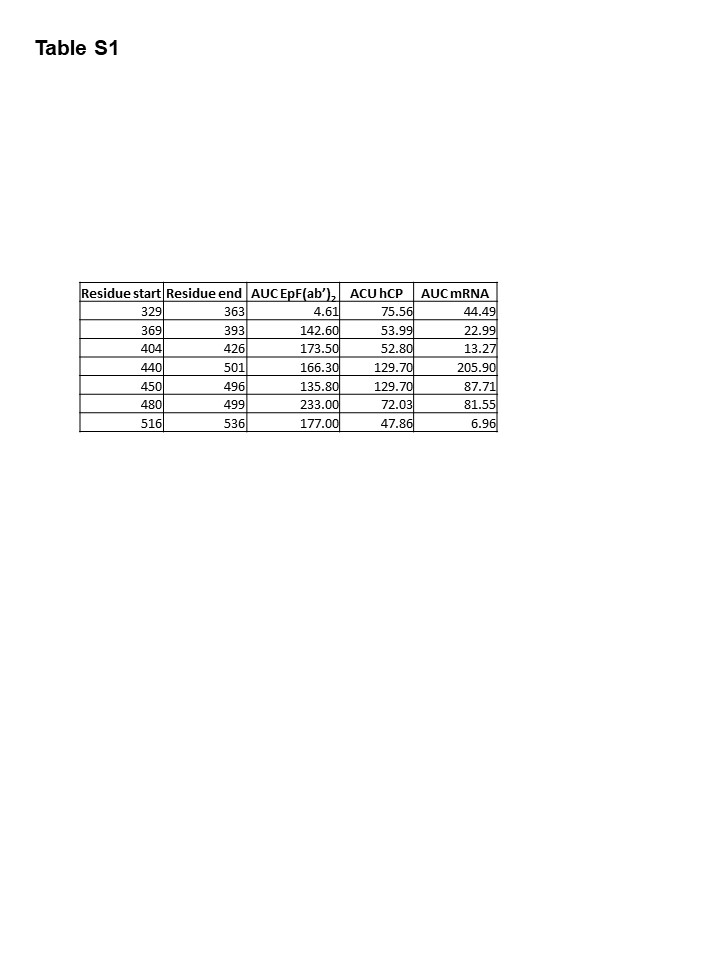

Supplement: Supplementary file 3 [file Image_3.jpeg]
